# Remarks on the Treatment of Fractures of the Lower Extremities without the Aid of Splints

**Published:** 1838-10

**Authors:** 


### Art. II.

*Remarks on the Treatment of Fractures of the lower Extremities without the Aid of Splints.* By J. F. Burke, m.r.c.s.—*London*, 1837. 8vo. pp. 39.

*A Practical Treatise on Fractures, illustrated with sixty Woodcuts.* By Edward F. Lonsdale, Surgeon, Demonstrator of Anatomy in the Middlesex Hospital School of Medicine.—*London*, 1838. 8vo. pp. 536.

We do not propose to give an analysis of the pamphlet of Mr. Burke, because it contains nothing which is not, to some extent, a matter of every-day practice; to as great an extent, indeed, as the principle can be safely applied. We apprehend the author's experience in the treatment of fractures must be very limited, or he would not have imbibed so great a horror of the judicious application of splints; a horror so intense, that he appears to deem any sacrifice light by which they may be superseded. By splints he can conceive nothing but an obdurate piece of wood or iron, which, if it enter not into the patient's soul, may at least very much inconvenience his limb. Now, in our innocence, looking at the subject in an enlarged sense, we had conceived that any apparatus by which the necessary resistance to displacement was offered, whether it were a piece of pasteboard, a junk, or any similar contrivance, was, in fracture-language, just as much a splint as a piece of board or iron so many inches long and so many wide. On this point we would adopt Mr. Lonsdale's sentiment:

> "It is the fact of many fractures getting well by position only that, I believe, has led some surgeons to adopt the erroneous opinion that splints are altogether useless, or any mechanical contrivance that tends to keep the ends of the bone in apposition. How far such an opinion is correct, any one who has had much experience in treating fractures must be able to judge; for there are many cases that absolutely require mechanical means to steady the portions of bone, while the majority of fractures are benefited by splints of some kind. I am inclined to think that those who advance the sweeping assertion of splints being useless, have formed their opinion upon very limited experience, and judge of single cases that may have got well under their care without their employment, instead of taking the majority of cases, which are always benefited by their use, and many of them absolutely requiring it." (p. 51.)

The impression we derive from the perusal of the second work, from which we have just quoted, is, that Mr. Lonsdale is a careful observer, that he possesses ingenuity in mechanical contrivances, and that he has a competent knowledge of the routine treatment of fractures in the hospitals of this country; but, if we had been consulted, we are not sure that we should have advised him to publish the work before us. It would require a better reason than the difference in price to convince us that, whilst we possess Sir A. Cooper's work, the profession required the present. This most distinguished surgeon, in the plenitude of his experience, felt it was unnecessary to give any other than his own ideas: but our author is a much younger man, his experience comparatively small, and, to render his work acceptable, he should have made it complete. We cannot say that this is exactly the case, although it contains much, and may be safely consulted by the young surgeon in his difficulties. We, in particular, desiderate some account of fractures of the cranium and of the vertebræ; subjects of the highest importance. We also regret that no allusion is made in this work,—nor indeed, we may say, in the generality of works upon Fracture,—as to what should be done when there is a vicious consolidation. Should an attempt be made to rupture it? Some persons have come to the conclusion that such cases should be left to themselves; but we think the facts adduced by Fabricius and Purmann, and more recently by Œsterlen, seem to prove that the rupture of the callus may be often done successfully. Œsterlen gives eight cases which establish the possibility of straightening the limb by extension made at the time pressure was applied at the deformed joint, even at the end of the fifth week. For the particulars of these cases, and the mode of operating, we refer to the original work.* Various modifications of the same general mode have been employed by other men, and with considerable success.

We also have some reason to complain that Mr. Lonsdale has not used sufficient diligence in ascertaining modes of practice even amongst ourselves. Much time and space are occupied in proving that, in fracture of the forearm, the palm of the hand should not be placed against the chest. This was scarcely necessary: the common practice is not so: the dictum of our own quondam instructor was, "bring the hand so that you can spit into it." Again, he should be aware that, although luxations of the wrist are uncommon, yet that they may exist. In our own country, up to a very recent period, luxation of the wrist was supposed to be not an unfrequent accident; fracture of the lower portion of the radius being mistaken for it. In France, for many years, a directly opposite opinion was maintained, based upon the great experience of Dupuytren: he, as well as Marjolin, regarded all cases of luxation of the carpus, described by observers, as cases of fracture of the inferior extremity of the radius. We think it excessively doubtful whether the accident be possible without fracture, as a consequence of a fall upon the palm of the hand; because the repulsion of the ground is entirely directed upon the inferior extremity of the radius, and not upon the ulna: neither do we believe that the fracture of the radius is likely to occur as a consequence of a fall upon the dorsum of the flexed hand. In the work of Cruveilhier (*Anatomie Pathologique*, 9e livraison,) may be found conclusive evidence of the occasional occurrence of such an accident.

It must not be supposed, from these strictures, that we do not think the work of Mr. Lonsdale creditable to him, or that it does not contain much that is good. The ordinary modes of treatment practised in our country are plainly described in it; its woodcuts afford considerable assistance towards comprehending points which descriptions might leave doubtful; and several ingenious apparatuses, of easy application, of the author's own invention, are given in it,—especially one for fractured jaw: finally, it is published at a moderate price. We extract from it the following interesting table, showing the comparative frequency with which certain fractures occur. It will be interesting to compare it with a table of the same kind, and on a still larger scale, and containing more particulars,

* Ueber das Kunstliche Weiderabbrechen fehlerhaft, &c.—Tubingen, 1828.

extracted from the records of the Pennsylvania Hospital, published in our last Number.

*Table of the comparative Frequency of Fractures in the different Bones, observed at the Middlesex Hospital, from Sept.* 1831 *to Sept.* 1837.

| | | Out-Patients. | In-Patients. | Total. | |
|---|---|---|---|---|---|
| Hand | Phalanges | 63 | 3 | 66 | |
| | Metacarpus | 50 | — | 50 | |
| Fore Arm | Both bones | 85 | 8 | 93 | 386 |
| | Radius, singly | 193 | 4 | 197 | |
| | Ulna, singly | 63 | 1 | 64 | |
| | Olecranon | 29 | 1 | 30 | |
| | Coronoid process | 2 | — | 2 | |
| Humerus | Shaft | 78 | 11 | 89 | 118 |
| | Neck | 13 | — | 13 | |
| | Condyles | 16 | — | 16 | |
| Clavicle | | 260 | 13 | 273 | |
| Scapula | Body of the bone | 3 | 5 | 8 | |
| | Acromion process | 8 | — | 8 | |
| | Neck of the bone | 2 | — | 2 | |
| Face | Ossa Nasi | 10 | 3 | 13 | |
| | Upper jaw | 1 | — | 1 | |
| | Lower jaw | 9 | 23 | 32 | |
| Thorax | Ribs | 270 | 87 | 357 | |
| | Sternum | — | 2 | 2 | |
| Pelvis | | — | 7 | 7 | |
| Thigh | Adults | — | 144 | 144 | |
| | Infants | 37 | — | 37 | |
| Patella | | — | 38 | 38 | |
| Leg | Both bones | — | 197 | 197 | 289 |
| | Tibia | — | 41 | 41 | |
| | Fibula | — | 51 | 51 | |
| Foot* | Os Calcis | — | 2 | 2 | |
| | Astragalus | — | 1 | 1 | |
| | Toes | 4 | 7 | 11 | |
| Skull | | — | 48 | 48 | |
| Spine | | — | 8 | 8 | |
| Total number, during six years | | | | 1901 | |

With these few remarks we dismiss Mr. Lonsdale's work, and shall devote the remainder of this article to the consideration of one particular mode of treating fractures, which has been of late years extensively used in other countries, and which, we doubt not, will, ere long, be largely tested in our own. We are the rather led to confine our notice to this point because the more common treatment employed in England is presumed to be familiar to all our surgical readers, and because we foresee that this treatment is about to undergo important modifications, from the greater or less adoption of the treatment *by the permanent or immoveable bandage*, which we are now about to consider.

We are most ready to admit that, carefully and judiciously employed, the treatment by splints, such as are in common use, ensures the integrity of the limb; but at the same time we cannot conceal from ourselves that serious inconveniences attach to this method: the patient is long

* "Fractures of the small bones of the tarsus and carpus are not inserted as distinct fractures in the hospital books, but come under the head of severe lacerations or bruises, the injury of the soft parts being the most important point: I have, however, seen many cases."

confined to his bed; he becomes extremely fatigued by the position which it is necessary to preserve; the digestive and other functions are impaired; and the general health may, and often does, greatly suffer. To obviate these pressing evils, and to enable the patient to change his position as often as it becomes fatiguing, or indeed to rise from his bed, a variety of means have been employed; and it is singular that, with the exception of Amesbury's apparatus, and certain modifications of it, such as that in use at the North London Hospital, which have, from time to time, been, to a limited extent, introduced in practice, no means of enabling the patient to rise early from his bed have been much employed in this country. Better success attended the attempts which were made in other countries to escape the inconveniences alluded to, and several forms of permanent immoveable apparatus have been among the means employed for this purpose.

When or where this system of treatment originated, it is not easy to determine; nor can we be sure that the idea, suggested to the mind of Geoffroy by an inspection of some relics found in Egyptian monuments, —that some such system existed in those ancient ages,—was correct: certain, however, it is, that in very early times many of the nations of the East, Arabs, Persians, and others, employed the system, which has continued to be used up to the present moment, and which seems to retain much of its original simplicity.

At the sitting of the Académie de Médecine, held 13th January of the present year, M. Sedillot presented a fracture-apparatus which he brought from Constantine, and which he had taken from a Turkish woman, whose arm had been fractured by the bursting of a bombshell. This apparatus was composed of thirteen "*nervures*" of palm, each one inch wide, two to three lines in thickness, and nine inches long, convex on one surface, plane on the other, and arranged along and secured to a portion of prepared sheepskin; a space of three or four lines being maintained between them. This apparatus was supported upon the members by means of cords of wool loosely applied, but susceptible of constriction by twisting pieces of wood under them until the necessary force is given: in fact, a species of tourniquet. So arranged, this apparatus is perfectly applicable to simple fractures; but, in the case of the poor woman referred to, it was compound, and required dressing: to accomplish this without removing the apparatus, a portion of two of the splints, with the skin which covered them, was removed. Many modifications of this apparatus appear to be in use among the different tribes, and they are all ingenious. Certainly the contrivance which they employ to give to the apparatus the necessary constriction is worthy of remark; for it does not expose the member to any shock, and completely answers the end.

Although, in our estimation, the means to which we invite attention may be incontestably superior, we cannot withhold our admiration of the address and ability displayed by men who, deprived of all the resources we possess, having at their disposal only pieces of wood, coarsely divided, skins of animals, and undressed cords of wool, treat so methodically such a serious lesion, without neglecting any of the indications which European art, despite of all its perfections, has only lately recognized: we mean, the necessity of well and conveniently maintaining a state of perfect rest until the consolidation is complete.

According to De Pouqueville, (*Voyage dans la Grèce*, 1820-1,) the ancient as well as the modern Greeks employed a permanent apparatus, which was applied soon after the receipt of injury, and which, with perfect security, enabled the patient to move the limb in any direction: for the purpose of consolidating the apparatus, which was not removed from the moment of its application to that of cure, a composition into which mastic entered largely was used. A similar system, though brought about by other means, is employed in Spain, where it would seem to have been introduced by the Moors; in Corsica, where it is accounted a surgical heresy to remove an apparatus before the consolidation is complete; in the Brazils; in Italy, where it has been modified, of late, by Assalini, who used moistened pasteboard as the agent; in Switzerland, where, according to Fodéré, is found a class of men called "Rébouteurs," whose business is to set broken bones and to reduce luxations, and who travel through Switzerland, the Alps, Savoy, Alsace, and the Vosges, in the practice of their art. In the Vosges there is a family named Valdajos, who for two centuries have transmitted from father to son the secret of treating such accidents. Their success is said to be remarkable, and their modesty to equal their celebrity. Valdajos, Fleurot, and the Jollans attribute their success entirely to the permanence of their apparatus, which is composed of moistened card-board, or osier splints, solidified by mastic or pitch.

In 1734, the ancient Academy of Surgery in Paris offered the following as a prize-subject: "To determine, in each kind of surgical disease, the cases in which it is desirable to dress frequently, and those where it should be rarely done." The essay to which the prize was awarded was that of Lecat, who, in speaking of fracture, says, "A fracture, if simple, when reduced, only requires to be maintained: it may be examined *once*, to see that there is no displacement or deformity." In the year 1768, a memoir was presented to the same Academy by M. Moscati, in which he described a mode of treatment which he had employed for the cure of several fractures: he made a kind of mould, by means of compresses and bandages, saturated with white of egg: this apparatus remained applied up to the moment of cure.

The system thus introduced was not employed, even by the French, until the time of the earlier campaigns of Napoleon. In the first campaigns of Prussia and Poland, where the stations of the grand army were inconvenient and distant, and where M. Larrey found much difficulty in transporting the wounded, especially those suffering from fracture, he employed the system extensively, and with great success: in fact, many of the wounded at the battle of Moskwa were removed into France completely cured, and without a single removal of the apparatus. M. Larrey asserts, that, "whatever may be the nature of the fracture, the apparatus ought to remain applied, without removal, until the union is complete, and the wound, if there be one, entirely cicatrized; that no thought need be taken of the fluids or purulent matter which may be exhaled from the wound, because, in depriving these solutions of continuity, of contact with air, we isolate them, on the one hand, from the humidity or insalubrious condition of the atmosphere; on the other, we spare the patient the pains of frequent dressings; we prevent the chance of motion among the fragments of bone, local irritation, erysipelas of the part, or inflammation."

The apparatus he employed for the lower extremity was composed of a linen cloth, of the necessary length; two junks, an inch and a half in diameter, somewhat shorter than the linen; a small thin mattress, or cushion, stuffed with oats, and of the length of the linen; a "talonnière," or heel portion; a conical cushion, six inches long, three wide, and at its base two thick; three six-tailed compresses; bandages, and resolvent liquid—a mixture of camphorated spirit, extract of lead, and whites of egg, beaten up with water. The mode of application is quite simple: the limb is carefully raised, reduction is made, and the apparatus is disposed in the following order: the ligatures, five or six, the linen several times double, the bandage; the limb is now placed on the bandage, which lies upon the linen. The linen must extend beyond the heel and the popliteal space; compresses, dipped in the discutient fluid, are smoothly placed upon the fractured point; the bandage, similarly impregnated, is now brought carefully around the leg; the heel-piece is placed under the tendo Achillis, its base corresponding to the heel; the cushions are placed laterally in the hollow of the leg, the longest externally; the junks are now brought on either side of the leg, and rolled in the free border of the linen, until they are on the level of the leg: the ligatures are then drawn, constriction being gradually made from the knee downwards, care being taken to avoid placing one over the fracture; a stirrup is placed under the plantar surface of the foot, and attached to some of the ligatures. Such is the apparatus, which does not require much longer time to apply than those which are ordinarily used. It requires more care, however, because it is destined to remain through the whole time necessary for the cure.

In the preamble of his German translation of the *Parallèle* of Roux, published in 1817, Froriep says, "I am astonished that the method of the Moors of northern Africa has not been used: it consists in surrounding the limb with an envelope of plaster." He thought that it would be necessary to cast it in many pieces, so that it might be easily removed, if necessary. The first experiments on the subject at the Berlin Hospital were made by Keyl, at the suggestion of Professor Kluge, director of the hospital, in 1828 and 1829. In 1829, Dr. Rauch, and afterwards Dr. Muttray, made it the subject of their inaugural theses; and, after the latter, we shall describe the manner in which Dieffenbach arranges his apparatus.* He prepares a box of the necessary capacity; the limb is rubbed over with some fatty substance, is properly extended, and the semifluid plaster is poured in in sufficient quantity to envelope the limb: at present, if the fracture be complicated, he envelopes only four fifths, or even less, of the limb, so as to permit of the necessary dressings.

A correspondence has lately appeared in the London Medical Gazette, in which the merit of originating this method has been claimed by Mr. Sweeting, for Mr. Bond of Glastonbury, and by Mr. Beaumont for himself: perhaps, if this statement meet the eye of the parties, they may think it unnecessary to prolong a discussion which cannot end in giving to either party the merit of the invention. The apparatus invented by Mr. Beaumont for receiving the leg and the plaster, and at the same time keeping up extension, is ingenious: it is in the museum of the College of

* Muttray, De Cruribus Fractis gypso liquefacto curandis.—Berlin, 1831.

Surgeons in London. In the late Polish struggles, a similar method to that of Dieffenbach was advantageously employed by M. Malcz.

In 1834, M. Seutin, chief surgeon of the Hospital Saint Pierre, at Brussels, charged with the care of a large number of the wounded at the siege of Antwerp, (many of whom suffered from fracture,) employed the method of Dieffenbach as a means of treatment; but he found that the material lost in tenacity what it gained in solidity; that it did not resist external shocks—that it was easily broken; thus losing the most precious advantage, that of permitting progression. He then employed the apparatus of M. Larrey, which possessed neither of these drawbacks; but he found in it another: the ingredients which enter into its composition are not always easily procured, and its removal is troublesome, and the patient sometimes complains of its weight. These circumstances induced him to seek some means by which the principle might be carried out in a manner more free from objection; and such a means was presented to him in *starch.* By means of starch an apparatus was arranged which was easily procured, and not costly: it acquires considerable solidity, and a tenacity which enables it to resist perfectly external shocks; and, when its removal becomes necessary, it is easily accomplished without the destruction of any portion of the apparatus. From that time he has treated all cases of fracture, not only in the hospital, but in his private practice, by this method, with the most complete success.

We have ourselves employed this mode of treatment with the most gratifying result in some cases where the tumefaction was considerable.

In the practice of M. Seutin, on each occasion the bandage has been promptly applied, and the patient has been, in the greater number of cases, raised up, and placed on crutches, as soon as the bandage was thoroughly dry. Persons have proceeded on their journeys at the end of three days from the application of the apparatus. Those living in the town, with simple fracture, are bandaged, looked after for four or five days, then discharged: at the end of five or six weeks they come again to the hospital; the apparatus is removed; they are found cured, and ready to return to their usual occupations.

Now, beyond the success of the treatment, this is a matter of first-rate importance. At present, with us, a tradesman to whom such an accident occurs is prevented from attending to his business; a traveller, on urgent business, must be detained at, perhaps, a country village where the accident may have happened; while, if our statement as to the efficacy of the treatment be correct, no such inconvenience need attach to either.

The mode of application of this apparatus is as follows: immediately after the reduction of the fracture, the limb is surrounded by compresses dipped in Goulard water; a common bandage is carefully applied over these, from the root of the toes to the knee, (supposing the leg to be the seat of fracture:) this bandage is then, by means of a brush, covered over with thick starch; then another bandage is applied, beginning at the knee and descending towards the foot: this is covered like the first, to which it adheres, except on either side of the tendo Achillis, where a little padding is applied. Four pieces of thick pasteboard are moistened and moulded upon the leg, before, behind, and on either side of the fractured point: these are secured by two other bandages, one passing from the heel to the knee, the other from the knee to the heel, and covered by starch as before. From two to four days occur before the apparatus is perfectly dry; and from this moment the patient may get upon crutches, a support being given to the affected foot by a stirrup around the neck.

On the 25th September, 1837, M. Velpeau presented to the Academy of Sciences a memoir in which is discussed the two prevailing systems of treatment. He maintains that, whatever may be the nature of the fracture, whether accompanied by tumefaction or wounds of the integuments, reduction should be immediately proceeded with: this being accomplished, the part is to be surrounded by compresses, and a bandage moderately firmly applied, extending upwards from the points of the fingers or toes to the superior extremity of the fractured limb: the bandage is then to be brushed over with starch, and so on. The compression, being equal and moderate throughout, sustains the tissues, without occasioning the slightest uneasiness: the patient may turn, move, and act in bed as if there were only simple contusion of the leg. He is no longer condemned to lie on his back for six weeks or two months: he may get up on the third day; he may, without inconvenience, sit on a raised seat, because the leg may be moderately flexed; he may walk with the assistance of crutches, the foot being sustained by a stirrup around the neck. M. Velpeau submitted to the inspection of the section of Surgery, patients who walked on the second, third, fourth, and sixth day after the occurrence of the accident. It is admitted by M. Seutin that the principle upon which his system is framed is a simple, but certainly a successful modification of that of Larrey. It has been employed by him in about two hundred cases, with the greatest success; by Velpeau; by a considerable number of surgeons in France and Belgium; and by ourselves, in cases in every respect confirming the statements made in its favour by the persons to whom we have referred.

We shall now shortly consider the objections which have been raised against the principle upon which the system is founded; and we shall hold that, at the present moment, the method is most perfectly carried out by the apparatus of M. Seutin.

The principal objections which have been raised against this method of treating fractures are the following: If complete immobility be so necessary to prevent motion at the fractured point, this, it is objected, is often not attained because from thirty to sixty hours are required for the complete desiccation of the apparatus, during which time motion may be permitted; and because, if, at the moment of application, the tumefaction be considerable, when that subsides, a large space will be left between the bandage and the limb; and there will be, therefore, no resistance to displacement. The answer which may be given to this objection is, that, when a fracture is reduced, it is not usually in the first two or three days that displacement happens; it is after this period, and when motion, involuntary or indispensable, is daily repeated, and the ordinary bandages have become loosened, that this occurs. Now, although humid, the starch apparatus maintains coaptation during the first three days, with at least as much firmness as the ordinary apparatus; that time passed, the danger of displacement is over; the pieces entering into its composition form a compact, coherent mass, exactly moulded upon the limb, adapting itself to all the inequalities of surface, and forming with it a perfect whole. A

displacement, according to the axis or to the circumference of the limb, is completely prevented, because the superior fragment is included in the same mould as the inferior, and both must together be carried in any direction which is given to the limb; for no separate portion or fragment can possess the power of moving without the motion of the whole.

If we move a lower extremity thus maintained, what happens? If an impulsion is given to the foot, the movement is transmitted to the leg, to the thigh, to the pelvis: no partial movement can occur, but the whole member must be borne in the direction of the impulsion, and no displacement of the fragments can have place, because all are moved in the same direction at the same moment.

Already many substitutes for starch have been proposed, with the view of removing the inconveniences with which the apparatus is charged. A composition of starch and alum, glue, pitch, and other substances, have been named. M. Lafargue has just detailed a case in which he employed a mixture of starch with gypsum in fine powder, in which the apparatus was perfectly firm in six hours. M. Velpeau at present employs, with the same view, dextrine.

With respect to the second point, as to the space which may be left between the bandage and the limb, it usually happens that it is not considerable; it is not produced until after many days; the work of consolidation is then somewhat advanced; the ends are included in a callus which affords a powerful support.

Supposing the apparatus to be placed on a limb which is much tumefied, and that tumefaction rapidly subsides, so that at the end of a few days a considerable interval is found to exist between the limb and the bandage, the inconvenience may be remedied by firmly applying another bandage. The solidity of the apparatus might be thought an obstacle to the action of the bandage, but experience has shown that the difficulty is not insurmountable.

Another objection which has been raised is, that the early application of a bandage, before the development of a coming tumefaction, may produce a strangulating effect. Experience has shown that the fears of gangrene, as a consequence of this pressure, are unfounded. When an ordinary bandage is too tightly drawn at the level of the fractured bone, whilst the lower or more distant portion of the limb is less firmly compressed, tumefaction follows, which, if the constriction be continued, will end in gangrene; but a pressure of an uniform character, extended from the toes or fingers, uninterruptedly, along the member, is opposed to the tumefaction of the parts and to gangrene: in fact, by such pressure, we may procure atrophy of the limb, but not gangrene. Therefore is it important that the compression should be exact and uniform; and, in a large majority of cases, its effects, so far from being injurious, will be found to be decidedly salutary.

In fact, there are two kinds of compression: the one which is opposed to the reflux of venous blood, (that, for instance, which we establish circularly around the arm when we wish to bleed a patient;) the other, which is opposed to the ingress of arterial blood and favours the egress of venous blood, is that of every agent which compresses a limb equally at all points, and commences by a methodical compression of the portion farthest removed from the trunk. The first determines gangrene when long continued; the second is decidedly opposed to inflammation: the first is as strongly repulsed by the advocates for permanent bandaging as by others; and the second must belong to that method properly employed. Still we would say that, where the tumefaction of the limb is very great, the application of the apparatus should be delayed for some days; not because pressure would exercise any baneful influence, but because, when the tumefaction is dissipated, too great an interval may be left between the limb and the bandage, and because some difficulty might be experienced in determining upon the exactitude of the coaptation.

Another disadvantage which has been attributed to this method, is, that the practitioner is left in the dark as to the state of the limb; that, when once applied, it must there remain up to the moment of consolidation of the fracture; and that, before this period, we cannot ascertain whether there be eschars, abscess, or other similar complications. If only moderate attention be used, none of these accidents need occur: they must produce pain and general symptoms; and nothing can be easier than to remove the bandage. As far as strangulation is concerned, too, the extremities of the fingers or toes, which are uncovered, will always give timely indication of the approach of gangrene.

Again, it has been said that the bandage may produce excoriation and eschars, in consequence of the unyielding nature of its materials, against which the integuments may rub and become ulcerated. When we consider that the soft parts within the bandage remain perfectly immobile, and that the compression is exercised equally at every point, the apparatus being exactly moulded on eminences and depressions, we shall conclude that the occurrence of such accidents must be extremely unfrequent. Besides, the first layer of starch is on the outside of the bandage, and not in contact with the skin, and, even were it otherwise, the inconvenience might easily be avoided by interposing between the bandage and the limb any substance which, without preventing the necessary compression of the parts, would obviate such an inconvenience.

Such are the objections which have been urged against the treatment of simple fractures by this method: against the system, as applied to the treatment of fracture with wound of the integument, a more formidable objection has been made. It has been stated that pus is confined, and that the consequences are extremely injurious. The most conclusive answer which can be made to this objection is, that many hundreds of cases have been treated by this method, and that such consequences rarely occur. When the tumefaction is dissipated, and the period of suppuration has set in, the pus is poured out, penetrates the compresses, and gradually the different parts of the apparatus become impregnated with it. Some time is occupied in the production of this effect, and a multitude of facts clearly demonstrate that, beyond this point, suppuration very rarely proceeds. At first the purulent matter occupies the space left vacant by the subsidence of the tumefaction, but its force of expansion proceeds no further; the compressive power of the apparatus restrains it; there is no reflux, and infiltration of the integuments hardly ever takes place. It is, therefore, a new obstacle to the farther pouring out of purulent matter, which soon undergoes many changes; certain fluid portions are absorbed; and, being preserved from contact with atmospheric air, it does not contract those deleterious qualities which, under the influence of this agent, it usually acquires: the other portion extravasated between the integuments and the apparatus, and which pervades the latter, evaporates, leaving a concrete portion, which forms a stratum around the limb, which singularly adds to the solidity of the apparatus.

Before we proceed further, we will refer to the apparatus of the Arabs; and, certainly, we cannot say less in its favour than that it is a true "immoveable" apparatus, of easy application, more simple and effectual than that of Dieffenbach; little less so than that of Seutin.

Those persons who have no fears with regard to the necessity of providing for the escape of pus, may urge the bad consequences of leaving the wound exposed, and of the less rapid cicatrization; and no doubt the introduction of that principle would be a most important improvement upon the Arab system. Certain it is their apparatus perfectly accomplishes the common indications in treatment; the fragments are maintained motionless, the member is submitted to a uniform compression, variable at will, without shock or disturbance: and, with respect to their arrangement when the fracture is compound, it must be admitted that, when the opportunity of examination is unfrequent, the course they take is the safest.

We will now contrast, very shortly, the two systems of treating fracture of the lower extremity:

The method commonly employed in this country requires a confinement to bed, in the same position, for a period varying between three weeks and as many months. If the patient be in robust health at the commencement of this treatment, it is damaged at the end; if he be debilitated, that debility is aggravated; if he be occupied in trade or any occupation requiring his personal attendance, it must suffer in his absence; if he be a traveller, where he falls there he commonly lies, whatever may be the inconvenience: if the fracture be compound, all these evils are aggravated; the patient may be pulled down by profuse suppuration, or his wound may be exposed to all the accidents to which such a surface is liable. There are also other considerations of much importance which belong to the subject: frequent watching is necessary, and this among the poor in rural districts is frequently impracticable. For instance, a man may be surgeon to a large union of parishes under the present administration of the poor-laws; three or four fractures may happen at different points of that union: it is impossible for him to give them all the necessary attention; and, though the fractured bone may unite, it is often at an angle. Under the other system, the necessary apparatus may be found in a cottage; it is of easy application; it is not irksome to the patient; tumefaction, when present, is more speedily dissipated, and, if it be not already developed, is almost constantly restrained; the patient may, under ordinary circumstances, be left with a conviction that all will go on well; and, when the union is accomplished, instead of being reduced, by confinement, to a debilitated condition, the patient is enabled, on the fourth or sixth day, to leave his bed; the tradesman to attend to his ordinary business; the traveller to proceed on his journey without risk; and, if the fracture be compound, the tendency to suppuration is restrained, and the injury rapidly reduced to one of a simple nature.

It is true that, in Mr. Amesbury's apparatus, we have long had a means of enabling the patient to rise early from his bed; but we have not usually availed ourselves of it; and, for this reason, we apprehend—though at last reason, if opposed to experience, must yield,—that an impression exists, based upon the analogies of wounds of the soft parts, that a depending position is not adapted to early consolidation. But the adoption of this system does not necessitate the depending position for a longer time than is perfectly comfortable to the patient; but it allows of the patient changing his position as often as he may please; rising from his bed, and moving without assistance, and without risk from one room to another. This might, no doubt, have been accomplished by means of Amesbury's apparatus, but it is cumbersome, costly, and much more fatiguing to the patient than that of Seutin.

To those persons who are unconvinced by our statements, we would say, make the experiment yourselves; make it fairly and without prejudice; and do not pronounce judgment without having experimentally tested its correctness.

There yet remains one method of treatment of sufficient importance to require that we should direct attention to it,—the method described by Mayor under the term hyponarthecia. This plan, suggested by M. Sauter, essentially consists in a square or oblong piece of board, longer than the fractured limb, a pillow destined to receive it, cords to be attached to the corners of this board, and a pulley by which it is to be suspended. Upon this apparatus the limb is placed, and secured by means of bandages, so as to form, as it were, one piece with the machine: if the bones are disposed to ride, extension is made, a bandage is applied on either side of the fractured portion to secure it.

In the *Gazette Médicale*, (1835, p. 433, and 1836, p. 187,) may be found propositions to modify this apparatus, made by M. Munaret: he proposes to substitute for the board a metallic gutter, not unlike that adopted by Mr. Liston.

The great object accomplished by these contrivances is to suspend the limb at any required elevation, which might vary with the exigencies of the patient, to admit of the application of dressing, and to enable the patient to be moved. But these desiderata are obtained at considerable sacrifice as compared with the immoveable system; but, compared with the system usually employed in our own country, its advantages are manifest.

So long, however, as the opinion is entertained that the consolidation at the fractured point is the result of an action by means of which a substance is poured out in and around the fractured extremities, and that that process is best accomplished by the most perfect repose, so long must we maintain the opinion that, the more perfectly this end is answered, the more easily and rapidly will the business be performed.*

* It would seem that, in the time of Cheselden, white of egg was employed by a quack in London, for the purpose of giving firmness to his apparatus.

